# *Heterocypris incongruens* maintains an egg bank in stormwater habitats and influences the development of larval mosquito, *Culex restuans*

**DOI:** 10.1002/ece3.10445

**Published:** 2023-08-22

**Authors:** Jacqueline Trujillo, Cameron D. Schwing, Ephantus J. Muturi, Carla E. Cáceres

**Affiliations:** ^1^ School of Integrative Biology University of Illinois Urbana‐Champaign Urbana Illinois USA; ^2^ Department of Evolution, Ecology, & Behavior University of Illinois Urbana‐Champaign Urbana Illinois USA; ^3^ USDA, Agricultural Research Service, National Center for Agricultural Utilization Research Crop Bioprotection Research Unit Peoria Illinois USA

**Keywords:** benthos, disease vector, ecology, temporary habitat, zooplankton

## Abstract

Dormant propagules can provide a rapid colonization source for temporary aquatic habitats and set the trajectory for community dynamics, yet the egg banks of stormwater management systems have received little attention. We asked which species hatched from the sediment of drainage ditches in Champaign County, IL, and found bdelloid rotifers and ostracods (*Heterocypris incongruens*) to be the most common taxa. These sites also are colonized by mosquitoes, and we established laboratory experiments to examine interspecific interactions between common co‐occurring taxa. *Culex restuans* larvae were reared in the presence or absence of *H. incongruens* at two intra‐ and interspecific densities (20 or 40 total individuals) and their survivorship to adulthood, development time to adulthood, adult body size, and sex ratio were determined. Survival for *Cx. restuans* was significantly lower at high larval density than at low larval density in both treatments. *Culex restuans* larvae reared in the presence of *H. incongruens* had a shorter development time to adulthood and emerged as larger adults compared to those reared in the absence of *H. incongruens*. The sex ratios in the *H. incongruens* treatments were female‐biased whereas those in the *Culex*‐only treatments were male‐biased. These differences may have epidemiological implications, as only female mosquitoes serve as disease vectors. Our results emphasize the importance of understanding interspecific interactions in influencing larval mosquito development traits.

## Graphical abstract

Dormant propagules can provide a rapid colonization source for temporary aquatic habitats and set the trajectory for community dynamics, yet the egg banks of stormwater management systems have received little attention. *Culex restuans* larvae were reared in the presence or absence of *H. incongruens* at two intra‐ and interspecific densities and their survivorship to adulthood, development time to adulthood, adult body size, and sex ratio were determined. Our results emphasize the importance of understanding interspecific interactions in influencing larval mosquito development traits.

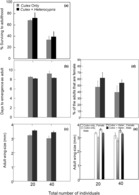

## 1 INTRODUCTION

Infrastructure designed to capture, control, and convey stormwater creates new habitats for freshwater organisms. This network consists of multiple structures, ranging from small catch basins that can hold <1 m^3^ of water, drainage ditches that are <1 m wide and only a few centimeters deep, dry detention basins that can be meters deep when full, to permanent retention ponds. Some of these structures, such as drainage ditches are designed to hold water for short periods following a rainstorm, but mismanagement can result in much longer hydroperiods, allowing for the development of a diverse community (Hassall & Anderson, 2015; Holmes & Cáceres, 2020). In addition to crustaceans, multiple mosquito species oviposit in these stormwater systems (Gardner et al., 2018; Mackay et al., 2016). The community composition of these aquatic habitats and the factors influencing the dynamics of these communities are just beginning to be described, hence, considerable work remains in determining the community ecology of stormwater habitats (Clifford & Heffernan, 2018; Sinclair et al., 2020).

In natural temporary aquatic habitats, community dynamics are driven by a combination of local and regional processes (Gabaldón et al., 2017; Márquez & Kolasa, 2013). One important process is rapid colonization from dormant propagule banks (Brendonck & DeMeester, 2003; Cáceres & Tessier, 2004; Rossi et al., 2004). Multiple species of rotifers, cladocera, copepods, ostracods, and insects produce dormant propagules at various life stages (eggs, larva, subadults, adults) some of which can survive for decades or centuries (Brendonck et al., 2017; Cáceres, 1997; Mergeay et al., 2007; Rossi et al., 2012, 2016). Both seasonal and prolonged dormancy can allow for rapid colonization of habitats following filling, and in some cases, influence the trajectory of community assembly (e.g., via priority effects Hairston Jr., 1996).

Once colonized, interactions within these systems are governed by the typical suite of interspecific interactions including predation, competition, and parasitism. Thus, quantifying which species that inhabit stormwater systems maintain dormant propagule banks, and how they influence the colonization of other species and the subsequent local interactions, is an essential component in understanding the ecology of these systems. *Culex restuans* (Theobald, 1901) is a known vector of West Nile virus in the Northeastern United States and one of the primary mosquito species that utilize stormwater systems for larval development. *Culex* and *Heterocypris incongruens* (Ramdohr, 1808) often co‐occur in these systems, which can be only a few centimeters deep, and both species are known to consume both algae and detritus (Delorme, 1991; Muturi et al., 2020; Rossi et al., 2011). Whereas spatial segregation is known to reduce interspecific interactions in many systems, the very shallow water column of temporary stormwater systems reduces the possibility of this separation.

Given the lack of knowledge of the community ecology of drainage ditches, we coupled field sampling of the egg bank with a laboratory experiment investigating the potential effects of intra‐ and interspecific competition between two common inhabitants of these systems, ostracod *Heterocypris incongruens* and larvae of the mosquito *Culex restuans*. We predicted that in these very shallow habitats, *H. incongruens* would compete with the larvae of *Culex*, reducing survivorship, increasing development time (time to pupation and time to adulthood), and reducing adult body size.

## 2 METHODS

### 2.1 Egg bank sampling

We sampled four sites in Champaign County, IL, USA (Table 1) to determine which species may be able to rapidly colonize drainage‐ditch communities from the sediment. On February 27, 2019, we used a tulip‐bulb planter (internal diameter: 5 cm) to collect five moist soil samples from each location. Each of the 20 samples was stored individually, uncovered in the dark at 4°C in plastic containers (700 mL) to mimic winter conditions for 4 weeks and to provide a refractory period (García‐Roger et al., 2006). In late March 2019, the temperature in the environmental chamber was increased to 20°C with a photoperiod of 14 h light: 10 h dark. We added 400 mL of aged filtered (glass fiber prefilter) lake water from Sportsman's Lake (Vermillion Co., IL) to each sample. Details of the water chemistry, which could influence hatching, are unknown. After a week, we siphoned the water from each sample through a mesh sieve (35 μm) to remove and record the number of hatchlings from the water. The water was poured back over the sediment sample, and the sediment was stirred to encourage emerging. All organisms retained on the sieve were identified using Delorme (1991) or Haney et al. (2013) and established in laboratory culture. We recorded emergence weekly for 4 weeks. Weekly counts were standardized to number of individuals per gram of sediment based on the final dry weight of sediment. Final dry weights were determined after drying the sediment in a drying oven and then subtracting the weight of the cleaned container.

**TABLE 1:** Location of the four drainage ditches that were sampled in Champaign County, IL.

Site	Latitude & Longitude	Taxon	2 April	9 April	16 April	23 April
1	40°5′3.6″ N 88°13′8.8″ W	Ostracods	0.15	0.64	1.10	0.76
		*Rotaria*	0.01	0.00	0.05	0.03
		*Brachionus*	0.01	0.00	0.00	0.00
		Springtail	0.00	0.00	0.00	0.00
2	40°5′48.3″ N 88°13′54.9″ W	Ostracods	1.21	0.16	1.08	0.97
		*Rotaria*	0.14	0.24	0.88	0.40
		*Brachionus*	0.00	0.02	0.03	0.00
		Springtail	0.00	0.00	0.00	0.00
3	40°6′45.9″ N 88°9′57.8″ W	Ostracods	0.02	0.09	0.10	0.16
		*Rotaria*	0.03	0.09	0.25	0.16
		*Brachionus*	0.00	0.00	0.00	0.00
		Springtail	0.00	0.00	0.00	0.00
4	40°6′55.6″ N 88°9′45.9″ W	Ostracods	0.00	0.00	0.00	0.00
		*Rotaria*	0.12	0.23	1.69	0.57
		*Brachionus*	0.02	0.03	0.01	0.01
		Springtail	0.01	0.01	0.01	0.01

*Note*: For each sampling date, the total number (summed across the five replicates) of each recovered taxa per gram dry weight of sediment is noted.

### 2.2 Ostracod‐mosquito interactions

We conducted a laboratory experiment to examine interactions between *Cx. restuans* larvae and *Heterocypris incongruens*. Egg rafts of *Cx. restuans* were collected from puddles on the campus of the University of Illinois, transferred to the laboratory, and allowed to hatch. Once hatched, to confirm species, first‐instar larvae were identified using the presence of a clear scale anterior to the sclerotized egg‐breaker (Reiskind & Wilson, 2008). All *Cx. restuans* were pooled together and were assigned to one of four treatments. We used our laboratory cultures of *Heterocypris incongruens* established from the egg bank samples as our source for the interacting species. Four treatments (five replicates each) were established to separate intra‐ from inter‐specific effects (10 *Cx. restuans* 1st instar larvae (hereafter “larvae”):10 *H. incongruens*; 20 larvae:0 *H. incongruens*; 20 larvae:20 *H. incongruens*; 40 larvae:0 *H. incongruens*). We selected these densities based on previous water‐column samples collected from these sites (Holmes, 2019). Turf grass infusion (23.94 g of grass per liter of tap water, soaked for 1 week) was used as the food source because the roadside ditches from which these species were collected received infusions of mowed grass. After a week of soaking, the grass infusion was filtered twice through a 105 μm sieve to remove the grass and other large debris. Each experimental beaker (400 mL Fisherbrand™ Tri‐Cornered Polypropylene Beakers) had a mixture of 20% grass infusion and 80% filtered lake water (70 mL grass infusion and 280 mL filtered lake water). Beakers were set in an environmental chamber at 25°C with a photoperiod of 14 h light: 10 h dark and stirred twice daily to prevent scum buildup. Any individuals that had pupated were removed during each visual inspection. We measured the survival of *Cx. restuans* and time of emergence to adulthood.

At pupation, mosquitoes were transferred to 50 mL tubes filled with 30 mL of distilled water. We examined each tube twice daily for newly emerged mosquitoes, which were killed immediately by freezing, and the time of emergence was noted. One wing was removed from each adult, mounted on a glass slide, and measured as a proxy for body size. We used the Leica Application Suite (LAS v4.10) to measure each wing to the nearest 0.1 mm from the jugal fold to the tip (excluding the fringe). Given that size differences among adults can be explained by competition and sex (females tend to be larger), the sex of each adult was also determined (Barr, 1958). The experiment lasted 17 days.

### 2.3 Statistical analysis

All data were analyzed using SAS software (Version 9.4, 2013). Each of the 20 sediment samples (five samples each from four sites) were incubated individually and sampled four times, but because of low rates of hatching, emergence numbers were pooled within the site on each date prior to analysis (Littell et al., 2002). A two‐way ANOVA was used to assess the effects of site and species on the rate of hatching per gram of sediment of the two dominant taxa. In the laboratory experiment with mosquitoes and ostracods, we used a logistic, two‐way ANOVA with a logit link in Proc Genmod to examine the role of density (20 vs. 40 per 350 mL), species combination (*Culex* only vs. *Culex* + Ostracod) and their interaction on survival to adulthood and sex ratio. We used two‐way MANOVA and the subsequent univariate ANOVAs to investigate the role of density, species combination, and their interaction on time to emergence and size at emergence.

## 3 RESULTS

**FIGURE 1:**
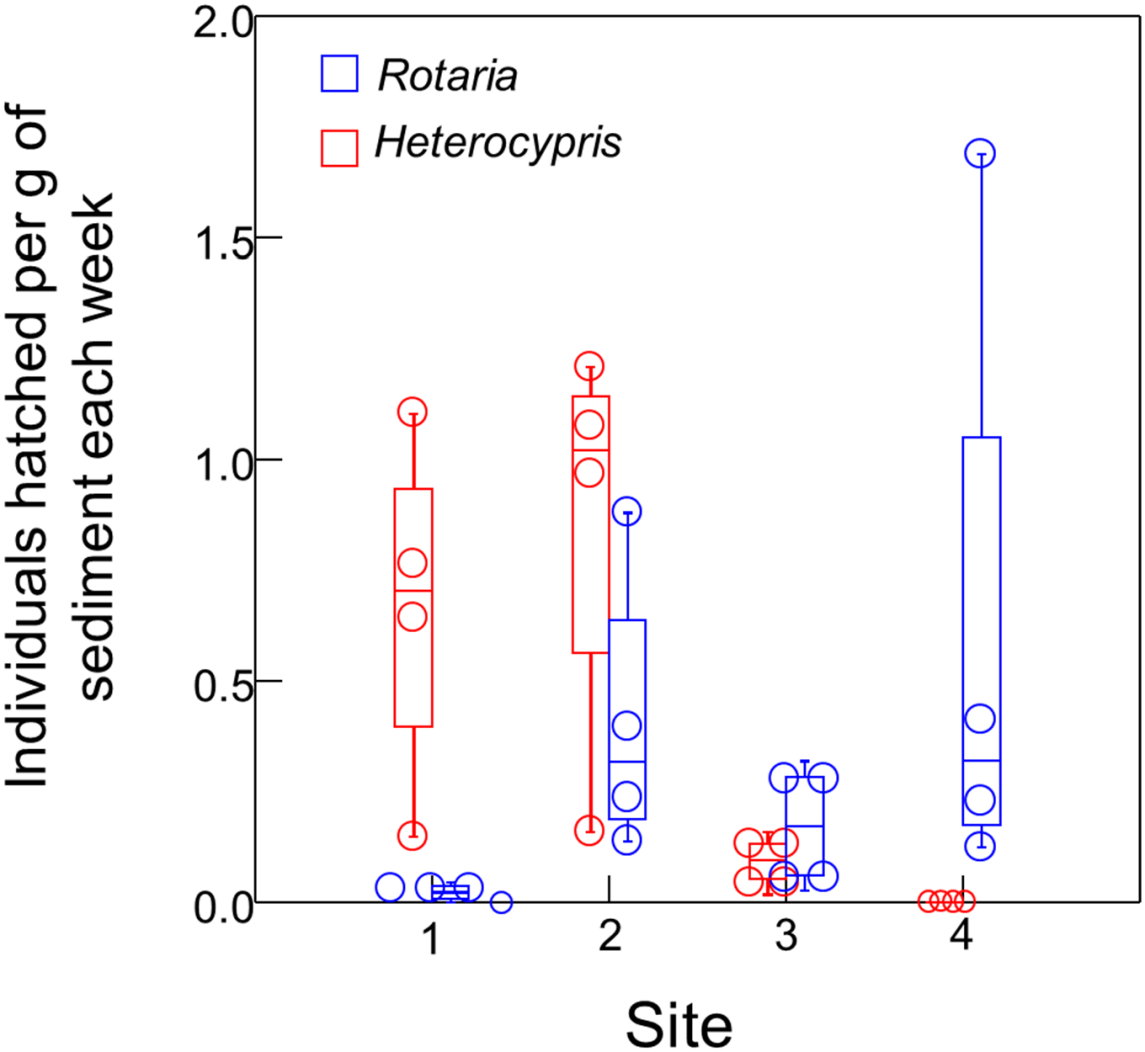
Emergence from the egg bank of the two dominant taxa (*Rotaria* and *Heterocypris*). The four dots in each box plot represent the 4 weeks of sampling. No ostracods were found at Site 4 and no *Rotaria* were found at site 1 during week 2 (smaller circles).

We found four taxa emerging from the sediments: Ostracods (*Heterocypris incongruens*), bdelloid rotifers (*Rotaria* spp.), monogonont rotifers (*Brachionus* spp.), and springtails (Collembola). Hatching continued for the 4 weeks of the experiment with 1387 individuals recorded from all four sites. For each sampling date, the total number (summed across the five replicates) of each taxa per gram dry weight of sediment is presented in Table 1. Springtails were the rarest taxa (7 individuals). Seventeen *Brachionus* were found across the four sites. *Rotaria* (594 individuals, 42%) and *Heterocypris* (769 individuals, 55%) were the most common taxa, with up to 100 individuals recorded per site on a single sample date (Table 1, Figure 1). All ostracods appeared to be *Heterocypris incongruens*, but each individual was not identified to species. For rotifers, the number recovered each date likely confounds new hatching and reproduction.

**FIGURE 2:**
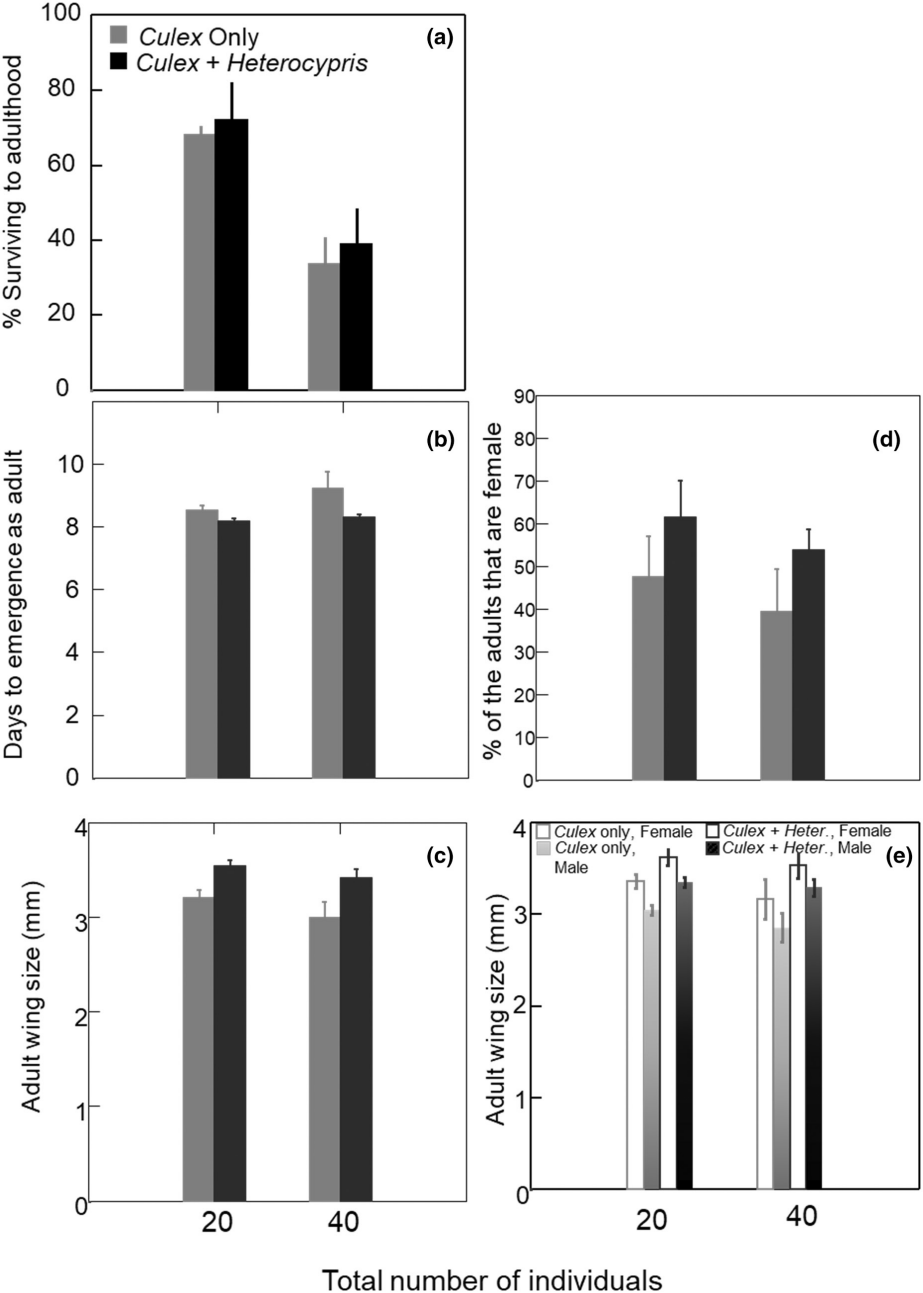
(a) The percent of mosquito larvae that survived to adulthood was influenced by density but not by the presence of ostracods. This difference in survivorship resulted in similar numbers of mosquitoes emerging from both density treatments. Time to emergence (b) and size at emergence (wing size) (c) were influenced by the presence of ostracods but not the initial density. (d) The presence of ostracods resulted in a female‐biased sex ratio in both density treatments. (e) The biased sex ratio could not fully explain the differences in wing sizes in the presence of ostracods; females were larger in the presence of ostracods.

In the laboratory competition experiment, the presence of *H. incongruens* and the density of total individuals had different effects on the four measured mosquito traits (survivorship, time to emergence, adult body size, and sex ratio; Figure 2). Doubling the total density from 20 to 40 individuals per 350 mL significantly reduced the survivorship to adulthood in *Cx. restuans* from ~70% to ~35% (Figure 2a, Table 2, *p* < .0001). As a result, the total number of adult *Cx. restuans* that emerged were similar in the low (20) and high (40) density treatments. In the *Culex*‐only treatments, three pupae died in the low density and four in the high density (total of seven) before reaching adulthood. No pupa died in the *Cx. restuans* and *H. incongruens* treatment. *H. incongruens* survival was high (>70%) in all but three replicates, with on average 80% ± 9.5% SE survival in the lower‐density treatment and 76% ± 13% in the high‐density treatment. We observed the reproduction of ostracods in one of the high‐density treatments. The presence or absence of ostracods and the interaction between mosquito larval density had no significant effect on mosquito larval survival to adulthood (Table 2).

**TABLE 2:** Results from the Multivariate ANOVA investigating the role of density, species combination (treatment – only mosquitoes or mosquitoes and ostracods) and their interaction on time to emergence and size at emergence and the subsequent univariate ANOVAs on both traits.

Logistic ANOVA survival	χ^2^ value	df	*p*
Density	41.55	1	**<.0001**
Treatment	0.89	1	.34
Den × Trt	0.01	1	.9

MANOVA	Pillai's trace	*F* value	df	*p*	Standardized canonical coefficients	
Factor					Wing length	Development time
Density	0.187	1.72	2, 15	.213	−0.859	0.585
Treatment	0.494	7.32	2, 15	**.006**	−1.263	0.132
Den × Trt	0.070	0.56	2, 15	.582	0.272	1.268

ANOVA time to adulthood	*F* value	df	*p*
Density	2.60	1, 16	.13
Treatment	5.83	1, 16	**.03**
Den × Trt	1.16	1, 16	.3

ANOVA wing length	*F* value	df	*p*
Density	3.00	1, 16	.10
Treatment	15.4	1, 16	**.001**
Den × Trt	0.17	1, 16	.68

Logistic ANOVA sex of adult individuals	χ^2^ value	df	*p*
Density	0.53	1	.47
Treatment	3.75	1	**.05**
Den × Trt	0.01	1	.95

3‐factor ANOVA wing length	*F* value	df	*p*
Sex	14.2	1, 32	**.0007**
Treatment	20.2	1, 32	**.0001**
Density	3.11	1, 32	.087
Den × Trt	0.59	1, 32	.45
Den × Sex	0.01	1, 32	.91
Trt × Sex	0.13	1, 32	.72
Den × Trt × Sex	0.01	1, 32	.91

*Note*: The results from the logistic ANOVAs for survival and sex at adulthood are also presented as are the results of the 3‐Way ANOVA for wing length. Bold values indicate *p* < .05.

The presence of ostracods, but not the initial density, influenced the three other traits (time to adulthood, adult body size, and sex ratio at adulthood). The MANOVA and subsequent univariate ANOVAs (Table 2) demonstrated that the presence or absence of *H. incongruens* was the primary driver of the differences in time to emergence and body size. Although the majority of mosquitoes emerged within the first 11 days, individuals from the high‐density, *Culex*‐only treatment continued emerging until day 17. The presence of *H. incongruens* significantly decreased the time to adulthood by more than half a day (Figure 2b, *p* = .03). Density and the density*treatment interaction had no effect (Table 2). In addition to emerging earlier, *Cx. restuans* that were raised in the presence of *H. incongruens* were larger than *Cx. restuans* raised only with other mosquitoes (Figure 2c; *p* = .001). Density and the density * treatment interaction again had no effect (Table 2).

Raising *Culex restuans* with *H. incongruens* influenced the sex ratio of the surviving adults. Eight out of ten of the replicates with *H. incongruens* were female‐biased (≥50% female), but only three of the ten *Culex*‐only replicates were female‐biased (Figure 2d, *p* = .05). These differences in sex ratio were similar at both larval densities (Table 2). Given that adult female mosquitoes are, on average, larger than males, we investigated the role of sex ratio differences in the observed difference in body size (Figure 2e). Female wings were on average 0.29 mm larger than male wings (*p* = .0007), but both males and female wings were on average 0.34 mm larger when they emerged from the treatment with *H. incongruens* compared to the *Cx. restuans* only treatment (*p* = .0001). Total population density had no significant effect on the mosquito's adult body size nor did any of the interactions (Table 2).

## 4 DISCUSSION

We combined field sampling of the sediment of four drainage ditches in Champaign County, IL with a laboratory experiment to explore interactions between larval *Cx. restuans* mosquitoes and *H. incongruens*. We first recorded the taxa that emerged from the sediment. Dormant propagule banks can provide a reliable colonization source in unpredictable environments (Cohen, 1966). Unlike natural temporary systems, where dozens of species have been found to emerge (Brendonck et al., 2017; Williams, 2006), we found only two common species in these drainage ditches, a bdelloid rotifer, and an ostracod. We predicted that competition between the ostracod *Heterocypris incongruens* and larval *Culex restuans* mosquitoes would result in decreased survivorship, slower developmental rates, and smaller body size for the adult *Culex*. We found that the presence of *H. incongruens* did not affect mosquito survivorship and had a seemingly beneficial effect on the other traits; placing *Cx. restuans* larvae with *H. incongruens* decreased the time to emergence and increased the body size, relative to the treatments containing an equal number of *Cx. restuans*. This increase in average body size was driven by both larger individuals overall (as determined by wing length), and more females (which are larger) being found in treatments containing ostracods. These females can serve as vectors of numerous human diseases including West Nile virus (Sardelis et al., 2001). As such, an understanding of how other community members influence the population dynamics of mosquitoes in these human‐created systems is needed.

### 4.1 Egg banks

Dormant propagule banks are common features of temporary aquatic systems (Brendonck et al., 2017). The size of the dormant egg bank is the result of variable annual contributions to the egg bank combined with differential survival and emergence (Bellin et al., 2020; Brendonck & DeMeester, 2003; Hairston Jr., 1996). We do not know which species, or how many of each species, were present in the egg bank since we only recorded what emerged. Our results are most likely influenced by the size of the egg bank, the resident species using different hatching cues, or some combination. Bet‐hedging strategies (not having all eggs hatch at once) are predicted to be higher in more uncertain habitats (García‐Roger et al., 2014; Rossi et al., 2012). The fact that emergence continued for 4 weeks suggests some degree of bet‐hedging, and/or differential exposure or response to hatching cues. Either way, during our experiment, the egg bank was not depleted, indicating that there are hundreds of dormant eggs per gram of sediment. These dormant eggs likely provide a colonization source each time the habitat fills and potentially sets the stage for community assembly. The two most common species we found are parthenogenetic, and both are well known to produce dormant eggs in other systems (Angell & Hancock, 1989; Bellin et al., 2020; Rossi et al., 2012). Bdelloid rotifers might have been so common in our samples because they can quickly terminate hatch from their dormant eggs and reproduce parthenogenetically between samplings. Given their small size and short life cycle with rapid reproduction, we did not investigate potential interactions between larval *Cx. restuans* and bdelloid rotifers.

### 4.2 Ostracod‐mosquito interactions

We demonstrated that the interspecific interactions of the *H. incongruens* produce equivalent results on survival compared to the intraspecific treatments. The effects of intraspecific competition among larval mosquitoes have been studied extensively, and survival is often reduced when larval density increases (e.g., Muturi et al., 2012; Ower & Juliano, 2019; Rowbottom et al., 2015; Westby & Juliano, 2017). Hence, the fact that increasing density lowered survival in our experiment is not surprising. However, the effects of interspecific competition on survival, time to emergence, and adult size, whether between two species of container‐dwelling mosquitoes or other organisms that share the same trophic level, varies based on the particular species involved and the exact environmental conditions (e.g., Costanzo et al., 2005; Grigaltchik et al., 2016; Juliano, 2009; Knight et al., 2004; Kroeger et al., 2013; Murrell & Juliano, 2008; Reiskind & Wilson, 2008). We found that the effects of interspecific competition are less than those of intraspecific competition on time to emergence and adult body size. This reduced competitive effect allowed the surviving *Cx. restuans* to emerge sooner at a larger body size. A potential reason for this may be spatial separation within the beaker between *H. incongruens* and *Cx. restuans*. Alternatively, the foraging rates of each *H. incongruens* maybe less than that of a larval mosquito and/or there is some mechanism by which larval resources are increased (nutrient recycling or suspension of benthic material).

Our results are contrary to other studies examining interspecific competition with species other than mosquitoes (see review by Blaustein & Chase, 2007) the majority of which examined competition between tadpoles and mosquitoes. For example, Mokany and Shine (2002) examined competition in two tadpole‐mosquito systems (*Limnodynastes peronii* and *Culex quinquefasciatus* and *Crinia signifera* and *Ochlerotatus australis*) and found that tadpoles in both systems reduced larval mosquito survival and growth rate. A few studies have demonstrated that ostracods and other crustaceans can compete for resources with mosquitoes (Blaustein & Margalit, 1991; Chase & Knight, 2003; Knight et al., 2004; Stav et al., 2005). For example, Rowbottom et al. (2015) explored the competitive effects between *Ae. camptorhynchus* and ostracods (*Diacypris* spp.) and found no effects on the larval survival or time to emergence, but that ostracods reduced adult body size. It is clear more studies on controphic competition are needed to establish the conditions under which interspecific competition will influence mosquito survival, time to emergence, and body size.

There are several potential explanations for the differences in sex ratios among treatments. The first is based on competition. Females require more resources to pupate (Ower & Juliano, 2019). Another possibility focuses on predation. *Heterocypris incongruens* is known to form predaceous swarms that prey upon small organisms such as mosquito larvae (Rossi et al., 2011). Although we did not observe any predation, our design does not allow us to separate competitive from predatory effects on mosquito survival. Male larvae are smaller than females. If the *H. incongruens* did attack the larvae, it is possible the smaller males were targeted in the treatments thus resulting in more females emerging. Finally, we assumed a 1:1 sex ratio at hatching for calculating relative survivorship since in *Culex* species, a 1:1 sex ratio is commonly observed (Clements, 1992; Ower & Juliano, 2019; Vinogradova, 2000). Sex‐specific deviations in hatching or alterations from 1:1 in the egg clutches could have also influenced our results.

Newly created aquatic habitats provide an opportunity to examine how the interaction of local processes such as competition, predation, and emergence from the egg bank interact with regional processes such as overland dispersal and oviposition to shape community dynamics (Jenkins & Buikema Jr., 1998; Louette et al., 2008). Clusters of stormwater habitats and the associated aquatic metacommunities may be particularly apt for such studies, as aquatic invertebrates vary in their dispersal abilities, their propensity to produce dormant propagules, and their life‐history strategies (Cáceres & Soluk, 2002). *Culex* mosquitoes are vectors for many human diseases, thus learning more about larval mosquito interactions with other coexisting species can aid with mosquito population control efforts. As natural wetlands continue to decline due to urbanization, these created habitats may serve as an important reservoir for aquatic biodiversity and expanding our knowledge on mosquito larvae ecology.

## AUTHOR CONTRIBUTIONS

**Jacqueline Trujillo:** Data curation (lead); funding acquisition (supporting); investigation (lead); methodology (equal); writing – original draft (lead); writing – review and editing (equal). **Cameron D. Schwing:** Conceptualization (equal); methodology (equal); writing – review and editing (equal). **Ephantus J. Muturi:** Conceptualization (equal); formal analysis (supporting); writing – review and editing (equal). **Carla E. Cáceres:** Conceptualization (equal); formal analysis (lead); funding acquisition (lead); resources (lead); writing – original draft (equal); writing – review and editing (equal).

## FUNDING INFORMATION

National Science Foundation grant DEB 1754115 and the Spyros Kavouras Memorial Undergraduate Summer Research Award through the School of Integrative Biology.

## Data Availability

The data will be deposited in Dryad https://doi.org/10.5061/dryad.dbrv15f4c.
